# Comparing statistical ‘phenomic prediction’ models for remote-sensing-based phenotyping of maize susceptibility to common rust

**DOI:** 10.1016/j.plaphe.2025.100134

**Published:** 2025-11-05

**Authors:** Johannes W.R. Martini, Osval A. Montesinos-Lopez, Jose Crossa, Rodomiro Ortiz

**Affiliations:** a International Maize and Wheat Improvement Center – CIMMYT, Mexico; b Facultad de Telemática, Universidad de Colima, Colima, Mexico; c Department of Plant Breeding, Swedish University of Agricultural Science, Alnarp, Sweden

**Keywords:** Phenomic prediction, Genomic prediction, Remote sensing, UAV, Common rust

## Abstract

We investigate the potential of phenomic prediction (PP) in remote-sensing-based phenotyping for genetic studies. Rather than relying on a single vegetation index, we utilize all available data collectively to predict the human-assigned visual score (VS). The conceptual motivation is that when a trained model is available, these predictions may provide a more accurate assessment of disease symptoms than the use of a specific vegetation index (VI). To evaluate the PP approach, we employ the predicted VS in a genome-wide association study (GWAS) and consider strength and position of the detected genetic signal. We use two different sets of predictor variables: i) the five basic wavelengths captured by a multispectral and a thermal camera (basic traits model, BT) or ii) all traits (AT), consisting of the five basic wavelengths plus ten vegetation indices. As statistical methods, we compare a) (linear) ordinary least squares regression (OLS), b) (linear) ridge regression (RR), c) (linear) least absolute shrinkage and selection operator (LASSO) d) an artificial neural network (ANN) and e) a gradient boosted regression tree method (GBRT). Our results indicate that the simple linear OLS regression on the five basic wavelengths (BT-OLS) performs on a level comparable to the best individual vegetation index G. The use of all traits in the OLS regression (AT-OLS) leads to overfitting, which was prevented by the regularization in AT-RR and AT-LASSO. The non-linear ANN approach seems to improve the results further, but the differences between the methods were not statistically significant. The strongest improvement for the purification of the genetic signal was observed when genomic estimated breeding values (GEBVs) for the different traits (VS, basic wavelengths, vegetation indices) instead of their adjusted phenotypes were used. Across all approaches, the combination of GEBVs with Ridge Regression or the non-linear ANN provided the best results.

## 1 Introduction

High-throughput phenotyping has significantly advanced agricultural research, particularly in the field of 'phenomics' [[1], [2], [3]]. In crop breeding, phenomic approaches use phenotypic data from non-target traits, like multispectral reflectance patterns or metabolite profiles, to help select for target traits, such as yield per hectare.

Analogously to genomic selection (GS) [[4], [5], [6]], where genome-wide markers are used, the term 'phenomic selection' underscores the simultaneous consideration of multiple (non-target) traits. This concept is different from marker-assisted selection (MAS) for molecular markers, or from 'phenotypic indirect selection' for which a few specific traits are considered which are known to be correlated to the target trait. The prediction of the target trait based on phenomic data (phenomic prediction, PP) utilizes patterns in the predictor variables to deduce similarities between individuals, which can be captured in 'phenomic relationship' matrices in mixed models [7] or other statistical methods [[8], [9], [10], [11]]. Like genomic prediction (GP), phenomic prediction does *not require* detailed biological understanding of why certain non-target traits predict a target trait effectively. In particular, the focus may lie on the overall predictions, not on the specific effects of features which is reflected in the common use of relationship matrices [8] condensing individual effects.

Examples of (genomic and) phenomic prediction include the work of Montesinos-Lopez et al. [[12], [13], [14]], employing Bayesian approaches to predict wheat grain yield, and Westhues et al. [[15], [16]] utilizing endophenotypes in linear mixed models to predict maize yield traits. Moreover, Roth et al. [17] suggested that non-target traits with reduced genotype ​× ​environment interaction could be useful in early generation selection decisions in wheat breeding. To manage large datasets, Runcie et al. [18] introduced 'MegaLMM', a software package for simultaneous mixed model analyses of many traits, and indicated high prediction accuracies by modeling genetic correlations among them.

Image data acquired from unmanned aerial vehicles (UAVs) have been instrumental in phenotyping disease symptoms in maize, particularly in cases such as the tar spot complex [19] and southern rust [20]. A prime example for a use-case for disease detection by remote sensing (RS) is to streamline disease monitoring in production fields to be able to apply field control measures at early stages of the disease progression. Recently, Loldaze et al. [21,22] examined the use of multispectral and thermal image data obtained from UAV-based RS for genetic evaluations in resistance breeding in maize, specifically for common rust, one of the most prevalent maize diseases [23,24]. The difference between applications for monitoring of production fields and for genetic evaluations is that the latter requires a more precise resolution of the severity of symptoms to base selection decisions on or to achieve better results in follow-up genetic analyses such as association studies.

**Fig. 1:**
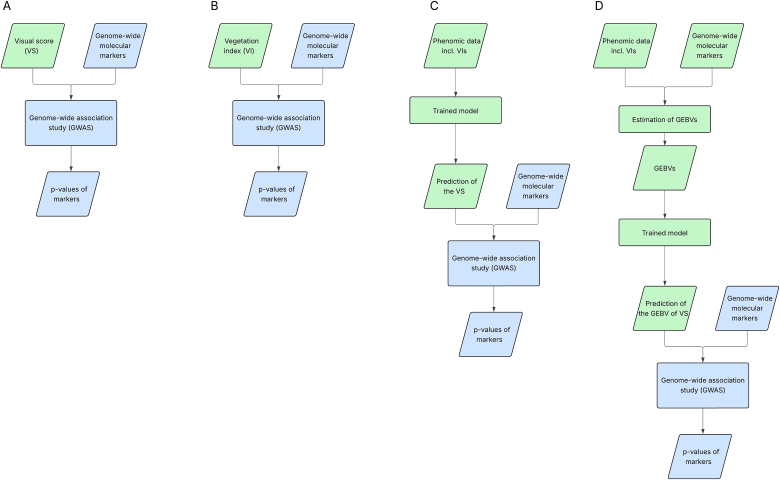
**Representation of different approaches for genetic evaluations.** Blue boxes represent the final genome-wide association study and are identical between the four approaches. The differences lie in the input of the response variable: A) the input is the visual score (VS) assigned by trained staff and adjusted for experimental design; B) replaces the VS by an individual specific vegetation index (VI) adjusted for experimental design; C) uses a phenomic prediction approach to predict the VS from phenomic data with a trained prediction model; D) instead of phenotypes adjusted to the experimental design only, genomic estimated breeding values (GEBVs) are used in the phenomic prediction of VS.

In Loldaze et al. [21], one of the objectives was to test an argument in favor of phenotyping by remote sensing, namely that it may be more objective than visual scoring by trained staff. Loldaze et al. [21] compared the genetic signal of genome-wide association studies (GWAS) when using RS-derived vegetation indices as trait (Fig. 1B) to when using a visual score (VS; Fig. 1A). The underlying idea was that a trait that is scored more objectively and that is more aligned with disease symptoms should lead to clearer and stronger signals in a genetic association study. This approach of using the strength of a detected genetic signal avoids to assume from the beginning that a specific phenotyping method is the better one. For the particular example of common rust, the genetic material that was used and the specific phenotyping methods, the authors identified the VS as having the highest quality for the purpose. However, RS data pointed to the same genetic region on chromosome 10 as being associated to resistance, indicating the potential of RS for high-throughput phenotyping. Among the vegetation indices, the simple reflectance ratio G ($\frac{R_{550}}{R_{670}}$; [25,26]) out-performed the other vegetation indices on average, but not for every considered case. Loldaze et al. [21] speculated that the superiority of the VS over all tested vegetation indices may be attributed to the staff's ability to discern common rust symptoms better from other influences which may also induce similar changes in multispectral reflectance.

The approach followed by Loldaze et al. [21] of comparing the performance of different individual vegetation indices with the aim to choose one of them for future RS-based phenotyping means following the concept of 'phenotypic (indirect) selection': A search for a specific trait that correlates well with the target trait 'susceptibility'. This raises the question whether a 'phenomic approach' -a statistical prediction based on all wavelengths and vegetation indices-could capture disease symptoms better. A statistical model can be trained to capture the relationship between predictor variables and disease symptoms and thus function as 'a tailored vegetation index'. Although prediction approaches come with other challenges, for instance around parameter fine-tuning to achieve a decent generalizability, such an approach may be beneficial for resistance breeding since vegetation indices were not specifically defined to capture disease symptoms, but mostly to capture more general plant characteristics (e.g. Ref. [27,28]). Moreover, the flexibility of statistical models would allow for relatively straightforward transfer and specification of the approach between different crops and diseases.

In this study, we investigate whether a phenomic prediction incorporating all available data (Fig. 1C), can outperform the best individual vegetation index (the simple reflectance ratio G, Fig. 1B). If successful, this approach could enhance future RS evaluations by using a trained phenomic model rather than a single index. This method is particularly valuable when the considered vegetation indices are ranked inconsistently across different datasets.

We use two sets of predictor variables, i) the five basic wavelengths (basic traits, BT) and ii) all traits (AT), that is the five basic wavelengths plus the ten basic-trait-derived non-linear vegetation indices provided by the data set, and apply five different statistical approaches: a) (linear) ordinary least squares (OLS), b) (linear) ridge regression (RR), c) (linear) least absolute shrinkage and selection operator (LASSO), d) an artificial neural network (ANN) and e) a gradient boosted regression tree (GBRT). OLS is the simplest regression method and prone to overfitting with increasing number of predictor variables. RR is a regularized linear regression approach and the standard statistical method in GP, where it is often referred to as ridge regression best linear unbiased prediction (RRBLUP) or genomic best linear unbiased prediction (GBLUP) when marker effects are rewritten as genomic relationship (For a history of BLUP in breeding see for instance [[6], [7],[29], [30], [31], [32], [33], [34], [35], [36], [37], [38], [39], [40]]). The LASSO approach [41] is an alternative regularization to RR which penalizes the absolute values of estimated effects instead of their squared values to cope with an increasing number of variables and collinearity between predictors. Compared to RR, LASSO will tend to estimate effects of small size rather as being equal to zero, which means excluding the respective variable from the model. The fourth method, the artificial neural network is state-of-the-art technology in machine learning and is in general anticipated to transform agricultural research and breeding [42,[43], [44]]. The last method, gradient boosted regression trees, is also a non-linear prediction method based on ensembles of decision trees where each new tree is trained to correct previous errors. The direct interpretability of parameters of both non-linear methods is difficult, but between the two methods, ANN and GBRT, the latter has some advantages concerning user-friendliness and a deterministic training of the model.

We train these models and use them on 'out-of-training-set' data to predict the VS. We then use these predictions as a response variable in GWAS to evaluate the method based on the enhancement of the genetic signal (Fig. 1C). As a final extension, we adjust phenotypic data with genomic data to obtain genomic estimated breeding values (GEBVs) of the traits which we use as input in the procedure described before (Fig. 1D).

## 2 Materials and Methods

### 2.1 Data

We use the data previously published by Loldaze et al. [21,22], which is available on the CIMMYT Research Data repository at https://hdl.handle.net/11529/10548898.

The dataset comprises both genotypic and phenotypic data from three F2-derived biparental doubled haploid maize populations. The three populations share the same susceptible parent DTMA-85.

The phenotypic data, that is ground truth disease data, as well as the remote sensing data is explained in the following in more detail. Additional information can be found in Loldaze et al. [[21], [22]].

#### 2.1.1 Ground truth disease data

Common rust is a major foliar disease of maize caused by the rust fungus *Puccinia sorghi* [[45], [46], [47], [48], [49], [50]]. The pathogen has a complex life cycle of different spore stages and causes lesions on leaves (UNL Cropwatch). The impact on agricultural production can be severe, common rust has for instance been listed as the 7th most destructive disease in the southern United States in 2017 [23].

The plant material was evaluated for CR resistance at the CIMMYT El Batan experimental station, close to Mexico City (Carretera México-Veracruz, Km. 45, El Batán 56237 Texcoco), at around 2200 m above sea level, in two years, 2019 and 2020. Trials included two replications of each genotype in a lattice design. Field plots were of dimension 2.5 ​m ​× ​0.75 ​m two-row plots.

A water-Tween 20 suspension of *P. sorghi* urediniospores was sprayed over two consecutive days on the plant material for inoculation. The time-point was approximately one month before expected flowering.

The visual scores were assigned by trained personnel approximately four weeks after the appearance of first symptoms. The symptoms were categorized on a 1 (very resistant) to 9 (very susceptible) scale. Since susceptibility was evaluated over two years, we can use six data sets given by the population-by-year combinations. Our analysis uses the phenotypes adjusted for the experimental design (Phenos_pop1_adjusted.txt, Phenos_pop2_adjusted.txt, Phenos_pop3_adjusted.txt), as well as genomic marker data (Loladze_et_al_genotypes_GID.txt.gz).

#### 2.1.2 Remote sensing data

Remote sensing was based on a fixed-wing eBee Plus platform (SenseFly Ltd., Cheseaux-Lausanne, Switzerland). A multispectral Parrot Sequoia camera (Parrot Drone SAS, Paris, France) measured reflection at 550 ​nm (40 ​nm full width at half maximum, FWHM), 660 ​nm (40 ​nm FWHM), 735 ​nm (10 ​nm FWHM), and 790 ​nm (40 ​nm FWHM). A thermal infrared camera, ThermoMAP (7.5–13.5 ​μm, Airinov, Paris, France) was used in separate flights. Flight altitudes were at 55 ​m above ground, around midday at sunny conditions. The ground resolution of the images was 6 ​cm for the multispectral camera and 12 ​cm for the thermal camera. A radiometrical calibration was conducted before each flight which was complemented by an adjustment for light conditions based on the incident light sensor. Flights were performed between one day before and one day after the VS evaluation. Orthomosaic processing was based on Pix4D Mapper® (v3.3.24; Pix4D, Lausanne, Switzerland) and then converted into reflectance/temperature data.

When accessing the data set, the wavelengths are referred to as gre (green), red, nir (near infrared), thr (thermal), and reg (red-edge). Moreover, there are ten vegetation indices, which are non-linear functions of the wavelengths (see Table 1 in Ref. [21]). The vegetation indices used were NDVI [28], RDVI [51], MSR [52], OSAVI [53], MCARI1 and MCARI 2 [54], TVI [55], GM1 [56], PSSRa [57] and G [26]. The closest wavelength response of the multispectral signal (consisting of 550, 660, 735, 790 ​nm) was considered to calculate the indices.

**Table 1:** **Summary of the models, analyses and results** and where the corresponding results can be found in more detail. The results presented are based on the 30 out-of training-set predictions, that is only on prediction when training and predicted set were different. Models were compared with two-sided binomial tests. Models that share a letter as superscript have not been distinguished at a 0.05 level. ST denotes a supplementary table.

Predictors	Stat. Model	Adjustment	Predictive ability				Performance in GWAS	
			times meeting or exceeding the benchmark	(average) Pearson correlation between prediction and target variable for out-of-training-set predictions	(average) Lin's concordance correlation coefficient between prediction and target variable for out-of-training-set predictions	Detailed results in	times exceeding the benchmark	Detailed results in
BT	OLS	Pheno	13	0.76^a^	0.74^a,b^	Table 2	14^a^	Table 3
AT	OLS	Pheno	2	0.41^b^	0.35^c^	ST2	–	–
BT	RR	Pheno	15	0.77^a^	0.74^a,b^	ST3	–	–
AT	RR	Pheno	18	0.77^a^	0.75^a^	Table 4	16^a,b^	ST4
AT	LASSO	Pheno	19	0.77^a^	0.75^a,b^	ST5	15.82^a,b^	ST6
AT	ANN-Relu	Pheno	15	0.77^a^	0.76^a^	ST7	16.88^b^	Fig. 2
AT	ANN-sigmoid	Pheno	11	0.76^a^	0.74^b^	ST8	–	–
AT	GBRT	Pheno	8	0.75^c^	0.71^d^	ST9	13^a,b^	ST10
AT	RR	GEBVs	19	0.79	0.78	ST11	26^c^	Table 5
AT	ANN-Relu	GEBVs	19	0.79	0.78	ST12	20.56^d^	Fig. 3
AT	GBRT	GEBVs	19	0.79	0.75	ST13	14^a,b^	Table 6

For its use in phenomic prediction, the data was centered to mean 0 and standardized to a variance of 1 [11].

### 2.2 Statistical models used to infer a 'phenomic vegetation index'

We investigate whether a phenomic prediction is preferable to the use of a single specific vegetation index when used for genetic evaluations of susceptibility.

#### 2.2.1 Ordinary least squares regression (OLS)

We use linear models of type

Eq. (1) VS=μ+Pβ+ϵ

where VS is the visual score of the respective population-by-year combination, μ is a constant vector giving the general mean, β the linear coefficients and P the phenotypes for different traits. Moreover, ϵ is a Gaussian distributed error vector with mean zero and variance $I\sigma_{\epsilon }^{2}$.

For P we used either only the five basic traits gre, red, nir, thr, and reg or all 15 traits, that is, the five basic traits and all the ten vegetation indices. P is therefore a matrix of dimension nx5 or nx15, depending on the set of predictor variables, and with n denoting the number of genotypes. We refer to the first setup as the basic traits (BT) model and to the latter as the all traits (AT) model.

Based on Eq. (1), the OLS estimator is

$${\left({\left(1,P\right)}^{\prime }\left(1,P\right)\right)}^{-1}{\left(1,P\right)}^{\prime }\;\mathrm{VS}$$

where the constant column vector 1 is added to capture the effect μ.

#### 2.2.2 Ridge regression (RR)

For ridge regression, β of Eq. (1) is considered random with independent and identical distributed entries $\beta_{i}∼N\left(0,\sigma_{\beta \;}^{2}\right)$.

The ridge regression estimator is

$${\left({\left(1,P\right)}^{\prime }\left(1,P\right)+\lambda \;I\;\right)}^{-1}{\left(1,P\right)}^{\prime }\;\mathrm{VS}$$

Where I is a diagonal matrix with 1 s on the diagonal except for the first entry which is 0. This is the case to capture that μ is modelled as a fixed effect.

We estimated the penalty weight λ as

$$\lambda =\frac{{\hat{\sigma }}_{\epsilon }^{2}}{{\hat{\sigma }}_{\beta }^{2}}$$

using the R [58] package regress [59,60] and a phenomic covariance matrix $PP^{\prime }$ in which each column, that is each of the traits, was centered and standardized to mean 0 and variance 1. Once $\hat{\beta }$ is estimated, RS data can be used together with $\hat{\beta }$ to predict the VS when evaluating a new experiment, rather than using an individual vegetation index.

#### 2.2.3 Least absolute shrinkage and selection operator (LASSO)

LASSO penalizes the absolute values of the coefficients (instead of the squared coefficients as RR). This 'small' difference to RR does not allow to represent it by a simple solver as for OLS and RR. We used the R-package glmnet [61] with the functions cv.glmnet(X,Y, alpha ​= ​1) to define the optimal value for the penalty weight λ and glmnet(X, Y1, alpha ​= ​1, lambda ​= ​λ) to estimate the coefficients of Eq. (1). The algorithm used by the package to determine λ is not deterministic and therefore the value slightly changes even when using the same training data. We therefore used 50 reps and report results based on averages.

#### 2.2.4 Artificial neural network (ANN)

For neural networks, many different architectures are possible. The focus of this paper was to explore the potential of methods with user-friendly software with almost default parameters. We used the R-package torch [62] and its function *nn_module* with 2 layers: 16 input variables (μ, 5 basic traits, 10 vegetation indices) which are activating 20 hidden variables which again define the output (self$fc1 <- nn_linear(16, 20) and self$fc2 <- nn_linear(20, 1)). We did not use a feedback but only a feedforward network. For the results presented here, we used the ReLU activation function (nnf_relu(x)). We also tested a sigmoid activation with the described parameters, but results did not improve. Some results using the sigmoid activation function can be found in Supplementary Table 8. As loss function the mean squared error (nn_mse_loss()) with Adam optimizer (optim_adam(model$parameters, lr ​= ​0.01)) was used. For the training procedure, 50 epochs were given. Model evaluation was done by model$eval() and model(). A complication of this method compared to OLS and RR is that the training (or its initialization, that is the definition of the starting weights) is not deterministic. Reproducibility can be achieved by fixing seeds (torch_manual_seed()).To account for the circumstance that with each training, one can obtain a slightly different model and consequently different predictions, we used 50 replications. Results shown will either show averages of the repetitions or a histogram, that is a distribution.

#### 2.2.5 Gradient boosted regression trees (GBRT)

For GBRT predictions, the R-package xgboost was used [63] which provides an implementation of the gradient boosting framework by Chen & Guestrin [64].

The model was trained using the training data in the function xgboost() with training data as xgb.DMatrix() and parameters max.depth ​= ​3, eta ​= ​0.1, nrounds ​= ​50, objective ​= ​"reg:squarederror", verbose ​= ​0, lambda ​= ​2, alpha ​= ​2, min_child_weight ​= ​3, gamma ​= ​1 and nthread ​= ​1.

The parameters (lambda, alpha, min_child_weight, gamma) were chosen as (2,2,3,1) based on a rough grid search on the integers from 0 to 10 and aiming at maximizing out-of-set predictive abilities when predicting phenotypic VS. Predictions were performed based on the test data and the function predict().

### 2.3 Using genomic estimated breeding values of the traits

The adjusted phenotypes provided by Loldaze et al. [21,22] have been adjusted for the experimental design, but not for genomic relationship. Since the plant material is closely related, one can consider the different genotypes as partial replications of each other. A further transition from phenotypic data to genomic estimated breeding values (GEBVs) could therefore improve data quality by separating genetic signal from noise.

We used the following model to adjust each phenotypic response per single trait and per population-by-year combination

P=μ+g+ϵ

with P being the phenotypic data adjusted to the experimental design as provided by Loldaze et al. [21,22] and standardized to mean 0 and variance 1, and which was used earlier as predictor variable and here as response variable. Moreover, μ is a constant vector providing an overall mean and ϵ an error. The central quantity g is the vector of GEBVs of the respective trait. The GEBV can be interpreted as ‘the phenotype adjusted to genomic relationship’ or as ‘the response variable phenotype regressed on the genomic markers’. We modelled $g∼N\left(0,\sigma_{g}^{2}K\right)$ and $\epsilon ∼N\left(0,\sigma_{\epsilon }^{2}I\right)$ with K=MM’/9051, the genomic relationship matrix, M the matrix of marker sores and 9051 the number of markers. We used the letter K here for the genomic relationship matrix to avoid confusion with the vegetation index G.

‘Marker scores’ refers to the values −1, 0, 1 reflecting the counts of a reference allele at the respective single-nucleotide polymorphism (SNP) from which the number 2 was subtracted. Thus, the value ‘-1’ refers to the homozygous state of the alternative allele, ‘0’ to the heterozygous state, and ‘1’ to homozygosity in the reference allele. Moreover, missing values were imputed (see Refs. [21,22]).

Furthermore, I is the identity matrix. The variance components $\sigma_{g}^{2}$ and $\sigma_{\epsilon }^{2}$ were estimated based on regress() and GEBVs $\hat{g}$ were obtained from the mixed model equations [[7], [29], [30]]. GEBVs of traits VS and G were used individually in a GWAS to test which effect the use of GEBVs has on the results. Furthermore, the GEBVs of all traits were used together in AT-RR and ANN and GBRT predictions. In this situation, the models were trained to predict the GEBV of VS, not the phenotype of VS.

### 2.4 Cross validation

The data set consists of six population-by-year combinations. In an application for plant breeding, the phenomic model needs to be trained before it can be used for new evaluations. If phenomic prediction would out-perform individual vegetation indices, the trained model would be used to phenotype susceptibility of germplasm in new experimental trials.

To mimic this situation, we trained the model on one of the six population-by-year combinations and used the trained model to predict the VS for the other combinations. In more detail, we used Eq. (1) for the OLS, the RR and the LASSO approach to estimate μ and β, and then used Eq. (1) together with the respective P of the other population-by-year combinations for predictions. For example, we used $VS_{1,2019}$ (the VS of population 1 in 2019) together with $P_{1,2019}$ to estimate ${\hat{\mu }}_{1,2019}$ and ${\hat{\beta }}_{1,2019}$ (by OLS or RR or LASSO). We then used the phenotypes $P_{2,2020}$ as inputs together with ${\hat{\mu }}_{1,2019}$ and ${\hat{\beta }}_{1,2019}$ to predict ${\hat{\mathrm{VS}}}_{2,2020}$**.** Finally, we investigated whether this prediction -which can be considered as a phenomic index derived from $P_{2,2020}$- is more strongly correlated to $VS_{2,2020}$ than any individual vegetation index.

The Pearson correlation of prediction and the target variable is usually referred to as ‘predictive ability’. As an additional measure for prediction accuracy, we also used Lin's Concordance Correlation Coefficient based on the implementation in the R-package epiR [65] as function epi.ccc(). As the final and central measure for how well a prediction is capturing the disease symptoms, we investigated its performance in follow-up GWAS, in terms of the size of the −log⁡(p) value of the strongest signal on chromosome 10.

Note, that for ANN and GBRT, the model is not based on Eq. (1).

### 2.5 Genome-wide association analysis (GWAS)

The genome-wide association study is used in this work to assess the quality of the phenotyping methods. If the quality of the phenotypic data increases by being more precise or more specific to the symptoms of CR, the genetic signal should be more clearly visible, and associated p-values should be smaller. The phenotyping methods compared are the VS, the vegetation index G, or phenomic predictions of VS by a previously trained model.

GWAS was based on the R package rrBLUP [37]. The function GWAS() was applied to the marker data together with trait data (for instance the VS, the vegetation index G, their GEBVs or phenomic predictions).

### 2.6 Overview of models used and their comparison via a binomial test

Table 1 provides an overview of the models and analyses described in the manuscript including the supplementary material. Overall, there are 2 different sets of predictor variables (BT/AT), 5 different statistical methods (OLS, RR, LASSO, ANN, and GBRT) which are based on phenotypes adjusted to the experimental design or GEBVs. Out of these 20 combinations, 10 are investigated here and a variant of ANN with sigmoid activation function was added (see Table 1).

For model comparison based on the cross-validation described above, we applied two-sided pairwise binomial tests using the R function binom.test(). When comparing two methods A and B, we considered the performance of the two models on a certain combination of training and prediction set, and defined it to be a success when B has reached a higher value, be it for Pearson correlation, for Lin's CCC or the −log(p) values from GWAS. We tested the Nullhypothesis that the probability of success is equal to 0.5. The alternative was that this success probability is not equal to 0.5 (two-sided test). Each of the 30 combinations in which training and prediction set were not identical were considered to be an independent draw of a Bernoulli distribution with the respective success probability. The methods were viewed as different when the p-value of the binomial test was lower than 0.05 (Table 1). The latter is reached in a two-sided test, when one method outperforms the other in less than 10 instances or in more than 20 instances out of the 30 cases in which training and prediction set are different. The models based on GEBVs were not compared to those based on adjusted phenotype with respect to predictive ability, since the predictions are targeting a different quantity, namely the GEBV of VS, not the VS itself. However, phenotype-based and GEBV-based predictions were compared together with respect to their performance in follow-up GWAS analyses. Whenever a one-sided binomial test was used for comparison this is mentioned specifically in the results. For a one-sided test with alternative ‘greater’, a significance level of 0.05 is already reached with more than 19 successes out of 30 drawings. For the non-deterministic models, averages across 50 repetitions were used.

## 3 Results

### 3.1 Absolute correlation of traits and VS

To define a benchmark, we first considered the correlation of the 15 traits with the VS in the respective data set. In five of the six population-by-year combinations, the trait G (green to red ratio) had the highest absolute correlation with the VS of the same population-by-year combination. Only for population 2 in 2019, the absolute correlation of NDVI was slightly higher. The correlations of highest absolute value are summarized in Table 2 in row ‘Benchmark’. Absolute values are given, but note that most indices, in particular G and NDVI have a negative correlation with the VS. The sign of the correlation is not relevant and could be changed by simply inverting the meaning of the 1–9 scale used for the VS. The vegetation index G has also been found previously to have the highest −log⁡(p) values in GWAS for five of the six population-by-year combinations [21]. The correlations can be used as a benchmark to see whether a phenomic approach can outperform the individual indices in terms of its correlation with the VS.

**Table 2:** **Predictive ability of BT-OLS for the VS.** Correlation of the BT-OLS predictions with the VS of the respective data set and when trained with the data of different population-by-year combinations. ‘Benchmark’ denotes the highest **absolute** correlation a vegetation index reached with the VS of the respective data set. Note that most vegetation indices exhibit a negative correlation with the VS, which reflects the orientation of the scale of the visual scores from 1 (very resistant) to 9 (very susceptible). Correlations equal to or higher than the respective benchmark are highlighted in bold. The benchmark correlation has been reached in 13 out of the 30 cases in which the training and prediction set were not identical. Lin's Concordance Correlation Coefficient between prediction and the VS is given in brackets.

	Predicting to						
Predicting from		Pop 1, 2019	Pop 1, 2020	Pop 2, 2019	Pop 2, 2020	Pop 3, 2019	Pop 3, 2020
	Pop 1, 2019	0.80 (0.78)	0.72 (0.69)	**0.78** (0.76)	0.77 (0.76)	**0.78** (0.77)	0.76 (0.75)
	Pop 1, 2020	**0.78** (0.73)	0.77 (0.74)	0.68 (0.65)	0.81 (0.80)	0.72 (0.68)	0.80 (0.79)
	Pop 2, 2019	**0.77** (0.75)	0.71 (0.66)	0.81 (0.80)	0.79 (0.76)	**0.79** (0.78)	0.77 (0.75)
	Pop 2, 2020	**0.70** (0.69)	0.72 (0.71)	**0.76** (0.74)	0.86 (0.85)	**0.76** (0.74)	0.85 (0.85)
	Pop 3, 2019	**0.78** (0.76)	**0.75** (0.71)	**0.80** (0.78)	0.82 (0.80)	0.80 (0.78)	0.82 (0.80)
	Pop 3, 2020	0.68 (0.65)	0.72 (0.71)	**0.74** (0.71)	0.85 (0.84)	**0.74** (0.71)	0.86 (0.85)
	Benchmark	0.69 (G)	0.73 (G)	0.74 (NDVI)	0.86 (G)	0.73 (G)	0.87 (G)

### 3.2 Phenomic predictions based on design-adjusted phenotypes

#### 3.2.1 Basic traits with ordinary least squares regression (BT-OLS)

The objective of this work was to investigate whether a phenomic approach, that is a trained model predicting a visual score (VS) from available data would provide more precise information on disease symptoms than any individual vegetation index does.

The first approach was to use the five basic wavelengths for each genotype in a linear model and to estimate $\hat{\mu }$ and $\hat{\beta }$ of Eq. (1) with an ordinary least squares regression. These parameters can then be used for a prediction based on the respective phenotypes P. The regression coefficients estimated from the different data sets are summarized in Supplementary Table 1.

The predictive ability of BT-OLS is summarized in Table 2. Out of the 30 possible predictions (excluding the case of the prediction set being identical to the training set), 13 cases led to a correlation with the VS that was equal to or higher than the benchmark correlation. This means that for these cases, the phenomic predictions obtained from a previously trained model are equally or higher correlated to the VS than the best single vegetation index is.

We then compared the results of GWAS when using this phenomic model trained on each of the population-by-year combinations to when the GWAS is based on the reference vegetation index G or the VS. The underlying idea of using GWAS for model evaluation is to investigate whether the genetic signal is enhanced.

The results are summarized in Table 3. The ‘phenomic’ OLS prediction on the basic traits outperformed vegetation index G in 14 out of the 30 cases, which also means that it did not outperform G in 16 out of 30 cases. Moreover, for all except for two cases, BT-OLS and vegetation index G identified the same marker as having the strongest association (Table 3, positions 2,954,643 bp, 2,639,580 bp and 20,858,205 bp, respectively).

**Table 3:** **Genetic signal in GWAS when using BT-OLS predictions as ‘phenomic’ vegetation index.** Genetic signals described by the highest -log(p) value on chromosome 10 and the position of the corresponding marker. Rows provide different traits, that is the VS, the vegetation index G and BT-OLS with varying training sets. Training of the model can be interpreted as defining a phenomic index. Cases in which training and prediction set are identical are highlighted as italic. Cases in which training and prediction set were different and the performance of the vegetation index G was outperformed by the BT-OLS model are highlighted as bold. The genetic signal obtained from vegetation index G has been outperformed in 14 out of the 30 cases in which the training and prediction set were not identical.

		Population 1		Population 2		Population 3	
		2019	2020	2019	2020	2019	2020
VS	Pos. −log(p)	2,954,643 24.77	2,954,643 23.69	20,858,205 15.98	20,858,205 20.10	2,954,643 33.32	2,954,643 29.33
G	Pos. −log(p)	2,954,643 12.16	2,639,580 6.88	2,954,643 14.75	20,858,205 11.40	2,954,643 14.19	2,954,643 19.04
BT-OLS Pop1 2019	Pos. −log(p)	*2,954,643* *17.29*	**2,639,580** **8.57**	**2,954,643** **16.39**	20,858,205 8.18	**2,954,643** **16.41**	3,909,507 12.66
BT-OLS Pop1 2020	Pos. −log(p)	**2,954,643** **16.83**	*2,639,580* *8.30*	**2,954,643** **15.83**	20,858,205 9.57	**2,954,643** **14.69**	2,954,643 14.01
BT-OLS Pop2 2019	Pos. −log(p)	**2,954,643** **14.76**	**2,639,580** **7.85**	*2,954,643* *16.52*	20,858,205 7.95	**2,954,643** **16.75**	3,909,507 13.13
BT-OLS Pop2 2020	Pos. −log(p)	2,954,643 11.69	2,639,580 5.44	2,954,643 13.49	*20,858,205* *9.23*	**2,954,643** **16.10**	2,954,643 17.07
BT-OLS Pop3 2019	Pos. −log(p)	**2,954,643** **15.81**	**2,639,580** **8.38**	**2,954,643** **16.92**	20,858,205 9.06	*2,954,643* *17.49*	2,954,643 14.19
BT-OLS Pop3 2020	Pos. −log(p)	2,954,643 11.03	2,639,580 5.07	2,954,643 12.89	20,858,205 9.12	**2,954,643** **14.83**	*2,954,643* *17.14*

#### 3.2.2 All traits with OLS (AT-OLS)

When extending the approach from the basic traits to all the 15 traits in an OLS regression, we observed the typical symptoms of overfitting (for details see Supplementary Table 2): When training and prediction set are identical, the prediction of AT-OLS is improved compared to that of BT-OLS, which means that the training set data has been captured better when more variables are present in the model. However, all values on the off-diagonal that is any ‘out-of-training-set’ prediction is less accurate (compare Table 2 to Supplementary Table 2). The correlation of prediction and VS of the respective data set only exceeded the benchmark for two out of 30 cases, namely predicting populations 1 and 3, 2019 from population 2, 2019. Also, in follow-up GWAS analyses, the approach failed and exceeded the −log(p) of G only in two of the 30 cases (data not shown). The fact that AT-OLS shows signs of overfitting indicates that regularized regressions based on shrinkage such as RR and LASSO may be valuable*.* These techniques are standard procedures when predictor variables exceed robust estimation capacity [66].

#### 3.2.3 Basic traits with ridge regression (BT-RR)

Ridge regression is a regularization method that can help to prevent overfitting, and it is the reference approach in literature related to genomic selection (GS). We applied ridge regression to the case of using the 5 basic wavelengths. The predictions reached or exceeded the benchmark in 15 out of the 30 cases (Supplementary Table 3). For the GWAS analysis, the −log(p) value of the vegetation index G was exceeded in 14 out of the 30 cases (data not shown). All in all, a clear improvement from BT-OLS to BT-RR was not observed.

#### 3.2.4 All 15 traits in a ridge regression approach (AT-RR)

We applied the ridge regression approach to the situation in which all 15 traits are used and for which the OLS regression showed strong signs of overfitting. For the prediction of the VS, this method reached or exceeded the correlation benchmark in 18 out of 30 cases (Table 4). Moreover, the −log(p) values in the GWAS analyses outperformed the vegetation index G in 16 out of the 30 cases (Supplementary Table 4). Concerning the location of the identified signal, in all except for five cases, AT-RR and vegetation index G identified the same marker as having the strongest association (Supplementary Table 4).

**Table 4:** **Predictive ability of AT-RR for the VS.** Correlation of the AT-RR predictions with the VS of the respective data set and when trained with the data of different population-by-year combinations. ‘Benchmark’ denotes the highest (absolute) correlation a vegetation index reached with the VS of the respective data set. Correlations equal to or higher than the respective benchmark are highlighted in bold. The benchmark correlation has been reached in 18 out of the 30 cases in which the training and prediction set were not identical. Lin's Concordance Correlation Coefficient between prediction and the VS is given in brackets.

	Predicting to						
Predicting from		Pop 1, 2019	Pop 1, 2020	Pop 2, 2019	Pop 2, 2020	Pop 3, 2019	Pop 3, 2020
	Pop 1, 2019	0.81 (0.80)	0.71 (0.68)	**0.78** (0.76)	0.73 (0.70)	**0.78** (0.78)	0.74 (0.71)
	Pop 1, 2020	**0.78** (0.76)	0.78 (0.75)	**0.76** (0.74)	0.84 (0.82)	**0.77** (0.76)	0.85 (0.84)
	Pop 2, 2019	**0.76** (0.74)	0.71 (0.69)	0.82 (0.80)	0.80 (0.78)	**0.79** (0.78)	0.76 (0.75)
	Pop 2, 2020	**0.71** (0.70)	**0.74** (0.73)	**0.76** (0.75)	0.87 (0.86)	**0.75** (0.75)	**0.88** (0.87)
	Pop 3, 2019	**0.78** (0.75)	**0.73** (0.70)	**0.81** (0.78)	0.82 (0.79)	0.81 (0.79)	0.80 (0.77)
	Pop 3, 2020	**0.69** (0.69)	**0.74** (0.73)	0.72 (0.71)	**0.87** (0.86)	0.72 (0.72)	0.89 (0.88)
	Benchmark	0.69 (G)	0.73 (G)	0.74 (NDVI)	0.86 (G)	0.73 (G)	0.87 (G)

#### 3.2.5 AT-LASSO: A variable selection approach

As an alternative to the ridge regression, we used a LASSO approach which penalizes the absolute values of the coefficients and which can be considered as a variable selection method. The implementation used for LASSO follows a non-deterministic approach to determine the penalty λ. We reported average results of 50. Similar to AT-RR, AT-LASSO average predictive abilities reached or exceeded the correlation benchmark in 19 out of 30 cases (Supplementary Table 5). Moreover, its −log(p) values exceeded those of VI G in the GWAS analysis in 15.82 out of the 30 cases on average across 50 repetitions (One specific instance of the results of GWAS based on VS predicted by AT-LASSO is given in Supplementary Table 6).

#### 3.2.6 Non-linear models: AT-artificial neural networks

**Fig. 2:**
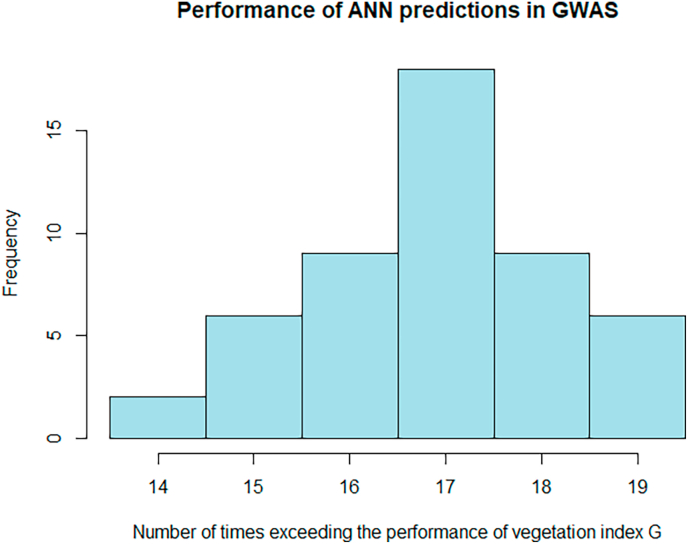
Histogram of the number of times that the predictions with ANN lead to higher -log(p) values than the vegetation index G in a GWAS analysis (and on chromosome 10).

Artificial neural networks are the state-of-the-art machine learning models and can outperform classical methods, in particular when the training data sets are ‘big’ or when hidden structures cannot be modelled explicitly as a component of a linear model [67]. With the implementation and parameters used in this work, the mean predictive ability for the VS exceeded the benchmark correlation in 15 out of the 30 cases (Supplementary Table 7). Recall here that the training algorithm has non-deterministic components which means that without setting a seed, obtained predictions will slightly differ. The mean predictive ability referred to here is therefore -as for LASSO- the mean correlation of 50 different training and prediction rounds. For the comparison based on GWAS, we considered as before the number of times the −log(p) derived from G is exceeded by the −log(p) value of the prediction across the 30 out-of-set predictions. Since the prediction is not deterministic, we investigated how this quantity behaves across 50 separate training and prediction rounds. The resulting histogram is shown in Fig. 2. The number reached from 14 to 19, with a mean of 16.88, which is slightly higher than what the other methods reached, but the improvement is neither clear nor on a relevant range.

#### 3.2.7 Non-linear models: gradient boosted regression trees (GBRT)

Compared to ANN, a simple advantage of the GBRT implementation based on xgboost is the deterministic training which gives more certainty for model evaluation. However, with the parameters used in this work and based on the phenotypic data, predictive ability for the VS exceeded the benchmark correlation in only 8 out of the 30 cases (Supplementary Table 9). Also, when the predictions were used in a GWAS analysis, the method outperformed the vegetation index G in only 13 out of the 30 cases (Supplementary Table 10).

### 3.3 Phenomic predictions based on genomic estimated breeding values (GEBVs)

#### 3.3.1 AT-RR based on GEBVs

The phenomic data used so far has been adjusted to the experimental design, but not to genomic relationship. A genomic relationship matrix allows to estimate the genomic estimated breeding value (GEBV) from the phenotypes and thus helps to dissect the additive genetic signal from the phenotypic data.

As a final step, we therefore utilized the genomic relationship matrix K to further adjust the phenotypic data (per individual trait and per population-by-year combination, see Materials and Methods), aiming to estimate a GEBV from the phenotypes.

We used the GEBVs of all traits, in an AT-RR approach. When predicting genomically adjusted ${\hat{\text{VS}}}_{K}$ by AT-RR based on genomically adjusted phenotypes ${\hat{P}}_{K}$, the predictive ability slightly increased compared to AT-RR without the genomic adjustment for predictors and the dependent variable (Table 1 and Supplementary Table 11). Recall in this context, that the predicted quantity differs (adjusted phenotype of VS or GEBV of VS). Additionally, the signals in GWAS were much stronger, and the approach outperformed the performance of the non-genetically adjusted vegetation index G in 26 out of the 30 cases (see Table 5). A binomial test counting successes when the phenomic AT-RR prediction based on ${\hat{P}}_{K}$ outperformed G, with Null-hypothesis of both being equally good (P(success) ​= ​0.5) and a two-sided alternative gives a p-value below $10^{-4}$. Concerning the location of the identified signal, the loci with strongest signal were identical to the position identified by vegetation index G in 28 out of 30 cases (Table 5).

**Table 5:** **Genetic signal in GWAS analyses when using AT-RR predictions based on the GEBVs of the traits instead of the provided phenotypes (AT-RR**${\hat{P}}_{K}$**).** Genetic signals described by the highest -log(p) value on chromosome 10 and the position of the corresponding marker. Rows provide different traits, that is the VS, the vegetation index G and AT-RR predictions when training a model on different training sets. Cases in which training and prediction set are identical are highlighted as italic. Cases in which the performance of the vegetation index G was exceeded by the AT-RR ${\hat{P}}_{K}$ method are highlighted as bold. The genetic signal obtained from vegetation index G has been outperformed in 26 out of the 30 cases in which the training and prediction set were not identical. Note that the GEBVs ${\hat{G}}_{K}$ and ${\hat{VS}}_{K}$ also lead to stronger genetic signals in the GWAS than their phenotypic counterpart of the same trait.

		Population 1		Population 2		Population 3	
		2019	2020	2019	2020	2019	2020
VS	Pos. −log(p)	2,954,643 24.77	2,954,643 23.69	20,858,205 15.98	20,858,205 20.10	2,954,643 33.32	2,954,643 29.33
G	Pos. −log(p)	2,954,643 12.16	2,639,580 6.88	2,954,643 14.75	20,858,205 11.40	2,954,643 14.19	2,954,643 19.04
AT-RR ${\hat{P}}_{K}$ Pop1 2019	Pos. −log(p)	*2,954,643* *25.44*	**2,639,580** **10.29**	**2,954,643** **17.72**	20,858,205 10.10	**2,954,643** **21.42**	2,954,643 17.29
AT-RR ${\hat{P}}_{K}$ Pop1 2020	Pos. −log(p)	**2,954,643** **20.69**	*2,639,580* *11.88*	**2,954,643** **17.70**	**20,858,205** **14.48**	**2,954,643** **19.90**	**2,954,643** **22.10**
AT-RR ${\hat{P}}_{K}$ Pop2 2019	Pos. −log(p)	**2,368,199** **19.14**	**2,639,580** **9.74**	*2,954,643* *23.52*	20,858,205 9.06	**2,954,643** **21.16**	2,954,643 16.66
AT-RR ${\hat{P}}_{K}$ Pop2 2020	Pos. −log(p)	**2,954,643** **16.09**	**2,639,580** **7.61**	**2,954,643** **16.55**	*20,858,205* *12.14*	**2,954,643** **16.08**	**2,954,643** **22.93**
AT-RR ${\hat{P}}_{K}$ Pop3 2019	Pos. −log(p)	**2,368,199** **17.76**	**2,639,580** **9.79**	**2,954,643** **17.30**	**20,858,205** **12.17**	*2,954,643* *19.86*	**2,954,643** **21.09**
AT-RR ${\hat{P}}_{K}$ Pop3 2020	Pos. −log(p)	**2,954,643** **16.54**	**2,639,580** **9.59**	**2,954,643** **15.54**	**20,858,205** **16.81**	**2,954,643** **17.88**	*2,954,643* *22.56*
${\hat{\text{VS}}}_{K}$	Pos. −log(p)	2,954,643 29.32	2,954,643 28.83	20,858,205 16.93	20,858,205 22.80	2,954,643 38.67	2,954,643 35.07
${\hat{G}}_{K}$	Pos. −log(p)	2,954,643 15.67	2,639,580 9.07	2,954,643 14.97	20,858,205 14.10	2,954,643 16.80	2,954,643 22.53

The observation of obtaining stronger genetic signals when using the GEBVs in AT-RR must be interpreted in the context of the general improvement when using GEBVs instead of the phenotypes, especially for the vegetation index G and the VS themselves (Table 5). The quantity that the models are trained to predict, that is the GEBV of the VS, provides a stronger signal than the VS itself. Moreover, when comparing the GWAS results based on AT-RR with GEBVs, to when based on the GEBV of the vegetation index ${\hat{G}}_{K}$, the first outperformed the latter still in 21 out of 30 cases. Therefore, the AT-RR model based on GEBVs outperforms here both, the adjusted phenotypes of G as well as its GEBV ${\hat{G}}_{K}$. The resulting model predicts more or less identical positions at a higher power than the (GEBV of) vegetation index G.

#### 3.3.2 AT-ANN based on GEBVs

**Fig. 3:**
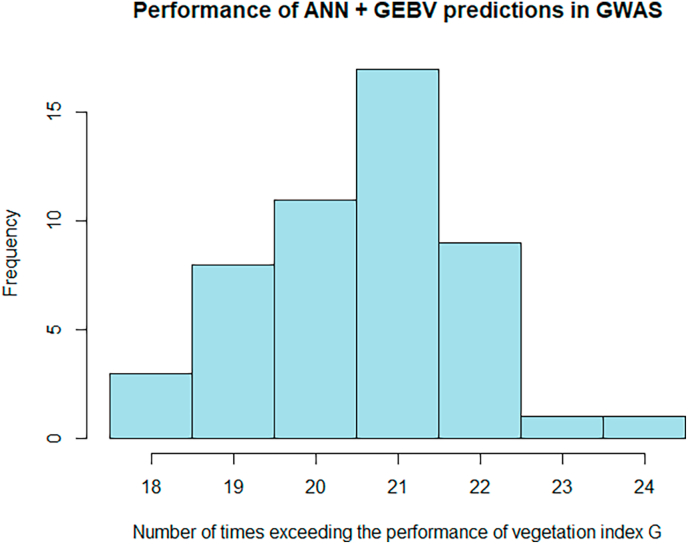
Histogram of the number of times that the predictions with ANN based on GEBVs lead to higher -log(p) values than the vegetation index G in a GWAS analysis.

**Fig. 4:**
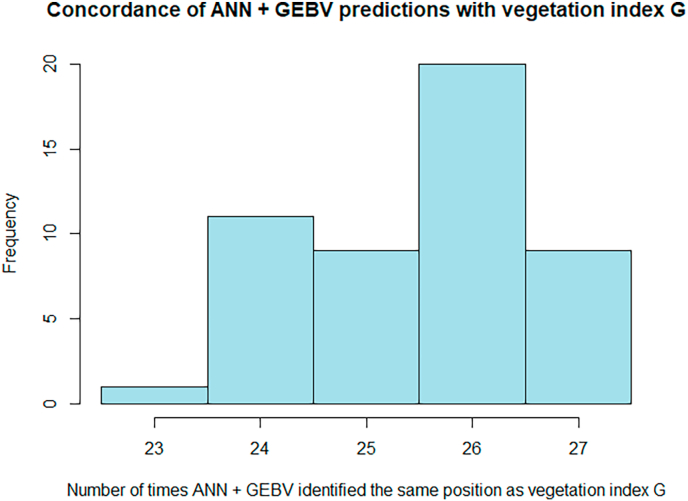
Histogram of the number of times that the predictions with ANN based on GEBVs identified the same molecular marker as having the strongest signal as vegetation index G.

Analogously to what we described for the AT-RR model above, we also applied the ANN to GEBVs. When predicting GEBVs ${\hat{\text{VS}}}_{K}$ by ANN based on GEBVs ${\hat{P}}_{K}$, the predictive ability was -as for AT-RR based on GEBVs-slightly increased (Table 1 and Supplementary Table 12). Also, the signals in GWAS were stronger, and the approach outperformed the performance of the vegetation index G in 20.56 out of the 30 cases on average (see Table 1 and Fig. 3). The small improvement from AT-RR to AT-ANN that was observed for the phenotypic data (from 16/30 to 16.88/30) is not visible when using GEBVs (26/30 compared to 20.56/30). When comparing to the GEBV of vegetation index G, ${\hat{G}}_{K}$, the ANN approach outperforms on average only in 10.26 out of the 30 cases (Supplementary Fig. 1). When applying binomial tests for model comparisons on the GWAS signals, AT-RR and AT-ANN with GEBVs were the only methods that clearly separate from all other methods (Table 1). Concerning the location of the signal, the AT-ANN model coincided on average in 25.5 of the 30 cases with the location identified by G (Fig. 4). Although, AT-ANN did not out-perform AT-RR when based on GEBVs, this approach of using ANN in combination with GEBVs may be a promising candidate for phenotyping by remote sensing in the context of resistance breeding. In particular also, because there may be potential for parameter fine-tuning of the model.

#### 3.3.3 GBRT based on GEBVs

The GBRT method did not perform well when phenotypic data was used, but responded to the transition from phenotypes to GEBVs: The predictive ability for the GEBV of the VS increased and the signals in the GWAS analysis gained strength. However, GBRT was still only able to outperform the VI G in 14 out of the 30 cases. Even though GBRT has the advantage of deterministic training compared to ANN, the observed performance would not support the claim to use it for the use-case tested here. Nevertheless, one should be aware that the method provides potential for parameter fine-tuning. The results observed here may also be a consequence of overfitting (see Supplementary Table 13).

### 3.4 Binomial tests for model comparisons

The two-sided binomial tests that were applied for predictive ability based on Pearson correlation could not show a significant difference between most phenotype-based methods (Table 1). The only methods that clearly separate from the others are the overfitting AT-OLS and AT-GBRT. The picture is similar when basing the tests on Lin's concordance correlation coefficient. Here, the models BT-OLS, BT-RR, AT-RR, AT-LASSO, AT-ANN separate into groups ‘a’, ‘b’ and ‘a,b’ (Table 1, Lin's CCC), but this is only due to the inclusion of the AT-ANN-sigmoid model. The most evident positive separation can be observed when considering the GWAS results and including the GEBV-based methods in the comparison. Here -as described above- the AT-RR-GEBV and the AT-ANN-GEBV model stand out on this data set (Table 1).

## 4 Discussion

### 4.1 From a single vegetation index to a phenomic prediction

Remote sensing is becoming increasingly important in high-throughput phenotyping, particularly in resistance breeding applications, where accurate and efficient prediction of plant traits is crucial. When exploring applications, a first step is usually to compare the performance of individual predefined vegetation indices [19,21,[68], [69], [70]]. These indices have been designed to capture certain physiological features of plants but do not necessarily describe the trait being studied precisely, in particular when dealing with specific disease symptoms. For instance, Thenkabail et al. [71] explored 490 channels in different narrow band normalized difference vegetation indices (NDVI) and their ability to fit the distribution of quantitative traits such as wet biomass, leaf area index, plant height and yield on a data set. The best wavelengths for NDVIs varied for the same trait between crops (for instance wet biomass between cotton and potato, Table 4 in Thenkabail et al. [71]), which may be interpreted as an argument to tailor the predictive model for a specific trait-crop combination. Thenkabail et al. [71] also used a linear regression approach together with variable selection restricting (mostly) to four variables, which they referred to as optimum multiple narrow band reflectance (OMNBR) and mention also the tendency to overfit when many variables are included simultaneously. These observations highlight the potential benefit of tailoring an index to the specific use-case, and set the stage for more complex models, that may involve statistical regularization, to be tested in out-of-set predictions.

In our study, we extended previous work on the use of remote sensing in resistance breeding [21] and shifted the focus from relying solely on pre-defined vegetation indices, that were motivated by other applications, to exploring the performance of 'indices' defined by statistical models in the context of resistance breeding. These models are aimed at predicting visual scores (VS) from phenomic data, and include a broader range of predictor variables, not only a single vegetation index. This approach may allow for a more flexible description of phenotypes, particularly in cases where the effectiveness of a specific vegetation index is unclear or inconsistent across different datasets.

Our analysis revealed that a simple ordinary least squares regression using five basic wavelengths (BT-OLS) was competitive with the best-performing vegetation index, G. This suggests that even basic models can be effective when predicting plant traits from phenomic data. In GWAS analyses, BT-OLS outperformed G in terms of the −log(p) value in 14 out of 30 cases, indicating that BT-OLS can be a simple alternative when it is unclear which vegetation index should be used.

When we incorporated all 15 traits simultaneously (five basic traits plus ten vegetation indices) using the AT-OLS model, we encountered signs of overfitting. Overfitting means that a model predicts well on training data but poorly on new data [72].This is evident in our findings where AT-OLS showed improved fitting for training data but reduced predictive accuracy for out-of-set predictions compared to BT-OLS.

To address the issue of overfitting, we employed regularization techniques. The ridge regression AT-RR improved both out-of-set predictions and GWAS signals slightly compared to BT-OLS. Comparing AT-RR to vegetation index G, the 16 out of 30 cases for which AT-RR outperformed G are not sufficient to declare AT-RR as superior, but it is worth mentioning that G was previously identified as the best vegetation index out of ten used by Loladze et al. [21].

The LASSO regression which was used as an alternative to RR, performed on a similar level (Table 1). Interestingly, the artificial neural network led to slightly better results in the GWAS although still on a level of little significance and relevance. The non-linear GBRT method did not stand out, neither in terms of predictive ability nor in terms of signal strength in GWAS, when phenotypic data was used.

### 4.2 Adjusting by the genomic relationship: The use of GEBVs for each trait instead of the phenotypes

Genomic Estimated Breeding Values (GEBVs) are a measure of genetic potential derived from genomic data. In contrast to phenotypic data, which is influenced by environmental factors or epistatic interactions, GEBVs are aimed at measuring the additive genetic contribution. Our study used the additive GEBVs for both the visual score and the RS data.

Typically, genomic and phenomic data are combined in predictive models using an approach such as

Eq. (2) Y=μ+g+p+ϵ

with both, the genomic term g and the phenomic term p being modelled as random (Y the phenotype, μ the overall mean and ϵ the error as described earlier). This model is challenging to interpret because it combines two variables, genomic and phenomic data, that will ‘overlap’ to a certain extend and will be difficult to separate. Moreover, when predicting new germplasm with such a model, a certain genetic relationship between training and prediction set is required to handle the term g.

We used the genomic data instead to pronounce the additive component of the phenotypes, which may potentially allow a better transferability to new germplasm in case the relation between additive component of phenomics and the target variable is general. From a conceptual point of view, the genetic relatedness between genotypes of training and prediction set could be lower than what is required for Eq. (2), since the genomic data can be used separately on training and prediction set to derive the GEBVs of phenomic data instead of including a joint genomic relationship matrix in the model. Only the genetic relationship within each set needs to be modelled, not the genetic relationship between training and prediction set.

In our analysis, using GEBVs instead of (adjusted) phenotypic data led to an improvement of the genetic signals in GWAS. Specifically, AT-RR outperformed the original vegetation index G in 26 out of 30 cases and AT-ANN outperformed G in 20.56 out of the 30 cases on average. This demonstrates that genomic adjustments improved GWAS signals in this study. The significance of this improvement is in particular visible for the VS and the vegetation index G themselves for which the use of the GEBV instead of the phenotype enhanced the signal in 6 out of 6 cases (compare G to ${\hat{G}}_{K}$ and VS to ${\hat{\text{VS}}}_{K}$ in Table 6). A binomial test with the Null hypothesis that phenotypic data and GEBV are performing equally and alternative that the GEBV has a higher probability to out-perform the phenotypic data, results in a p-value of 0.016, indicating that it is unlikely to observe such a 6 out of 6 result when there is no systematic effect. This improvement can be explained by a purification of the additive component of both the predictor as well as the dependent variable in the regression (which again could also be considered as a first ‘linearization’ of the data, and potentially as a preparation step for the linear GWAS).

**Table 6:** **Genetic signal in GWAS analyses when using GBRT predictions based on the GEBVs of the traits instead of the provided phenotypes (AT-GBRT**${\hat{P}}_{K}$**).** Genetic signals described by the highest -log(p) value on chromosome 10 and the position of the corresponding marker. Rows provide different traits, that is the VS, the vegetation index G and AT-GBRT predictions when training a model on different training sets. Cases in which training and prediction set are identical are highlighted as italic. Cases in which the performance of the vegetation index G was exceeded by the AT-GBRT ${\hat{P}}_{K}$ method are highlighted as bold. The genetic signal obtained from vegetation index G has been outperformed in 14 out of the 30 cases in which the training and prediction set were not identical. Note that the GEBVs ${\hat{G}}_{K}$ and ${\hat{VS}}_{K}$ also lead to stronger genetic signals in the GWAS than their phenotypic counterpart of the same trait.

		Population 1		Population 2		Population 3	
		2019	2020	2019	2020	2019	2020
VS	Pos. −log(p)	2,954,643 24.77	2,954,643 23.69	20,858,205 15.98	20,858,205 20.10	2,954,643 33.32	2,954,643 29.33
G	Pos. −log(p)	2,954,643 12.16	2,639,580 6.88	2,954,643 14.75	20,858,205 11.40	2,954,643 14.19	2,954,643 19.04
AT-GBRT ${\hat{P}}_{K}$ Pop1 2019	Pos. −log(p)	*2,954,643* *21.87*	**2,639,580** **8.40**	**2,954,643** **17.10**	20,858,205 9.43	**2,954,643** **18.35**	2,954,643 17.54
AT-GBRT ${\hat{P}}_{K}$ Pop1 2020	Pos. −log(p)	**2,639,580** **12.57**	*2,639,580* *13.69*	**2,954,643** **15.39**	2,017,344 9.83	**2,954,643** **14.21**	2,954,643 17.10
AT-GBRT ${\hat{P}}_{K}$ Pop2 2019	Pos. −log(p)	2,639,580 11.03	2,639,580 6.52	*2,954,643* *17.79*	2,017,344 7.42	**2,954,643** **16.25**	20,858,205 14.14
AT-GBRT ${\hat{P}}_{K}$ Pop2 2020	Pos. −log(p)	2,639,580 11.26	3,909,507 4.42	**2,954,643** **15.23**	*20,858,205* *15*.*20*	2,954,643 13.94	**2,954,643** **20.29**
AT-GBRT ${\hat{P}}_{K}$ Pop3 2019	Pos. −log(p)	**2,639,580** **12.68**	2,639,580 3.97	**20,858,205** **14.87**	2,017,344 9.00	*2,954,643* *25*.*21*	2,954,643 16.99
AT-GBRT ${\hat{P}}_{K}$ Pop3 2020	Pos. −log(p)	2,954,643 11.73	2,639,580 4.46	**2,954,643** **15.37**	**20,858,205** **12.77**	**2,954,643** **15.12**	*2,954,643* *24.24*
${\hat{\text{VS}}}_{K}$	Pos. −log(p)	2,954,643 29.32	2,954,643 28.83	20,858,205 16.93	20,858,205 22.80	2,954,643 38.67	2,954,643 35.07
${\hat{G}}_{K}$	Pos. −log(p)	2,954,643 15.67	2,639,580 9.07	2,954,643 14.97	20,858,205 14.10	2,954,643 16.80	2,954,643 22.53

Among the methods considered, RR and ANN were able to benefit from this enhanced precision. It is unclear why the GBRT approach was not able to capitalize on the GEBV data as the other methods did, but it may be related to overfitting (Supplementary Table 13), which points to parameter fine-tuning. The use of either RR or ANN, together with GEBVs resulted in models that identified the same marker as vegetation index G, but with a higher power. A downside of ANN is that the training is not deterministic and thus predictions change when the model is trained a second time with the same data. Overall, the use of GEBVs instead of adjusted phenotypes has been of value for the considered data set. The non-linear methods ANN and GBRT provide potential for fine-tuning of the parameters, but the RR provides the simpler, straight-forward approach which also performed best in this study when combined with GEBVs.

### 4.3 Relation to other recent work

Relatively close to our work on hand, DeSalvio et al. [20] applied different machine learning techniques to a use-case of Southern rust prediction based on 36 vegetation indices. The authors focused on the comparison of different machine learning approaches and temporal development using an RGB sensor with three basic channels. In contrast, our work neglects the temporal aspect, but extends to the use GEBVs of phenomic data, and assesses the different approaches in the light of the use-case of genetic evaluations by association studies. A result of DeSalvio et al. [20] intersecting with ours was the low performance of the ‘linear model’ on all considered traits, which can be interpreted as being analogue to our AT-OLS prediction.

### 4.4 Limitations

Our study was focused on a specific application of phenomic prediction in evaluating susceptibility to common rust. The analysis was conducted on three biparental DH populations with a shared susceptible parent. How a direct transfer of a trained model between unrelated populations would perform is unknown, but the modeling framework is flexible and can be directly trained on genetically more diverse populations. The combination of ANN with GEBVs showed good performance in our example and ANN methods have several parameters that can be fine-tuned. This property provides opportunities for fine-tuning but also poses the thread to overfit training data. Identifying the right set of parameters may not be obvious.

## 5 Conclusion

Our study i) emphasizes the ‘phenomic approach’ for experimental trial evaluation by remote sensing, ii) underscores the role that genomic data and Genomic Estimated Breeding Values (GEBVs) can play in phenomic prediction, for instance by enhancing data precision, and -although the linear ridge regression performed best- iii) suggests that neural networks may be able to capitalize on increased accuracy to improve results further when parameters are fine-tuned.

We employed a trained linear model as a ‘phenomic vegetation index’ for out-of-training-set data, rather than relying on a specific vegetation index. We demonstrated that a linear ordinary least squares regression using five basic wavelengths as predictor variables (BT-OLS) yielded results comparable to those of the top-performing vegetation index, G. Furthermore, our results suggest that incorporating all available traits into a linear ridge regression model (AT-RR), an artificial neural network (AT-ANN) or other non-linear methods may provide an advantage over using a single vegetation index. A phenomic approach may in particular be relevant when the effectiveness of different vegetation indices varies between experiments, and the integration of all available data may therefore offer a more robust solution than relying on a specific vegetation index.

The enhancement of GWAS results was particularly evident when using GEBVs of the traits instead of phenotypic data. This improvement was systematic and especially visible for both single-trait approaches based on the visual score (VS) or the vegetation index G for which the GWAS signals were enhanced for each of the six population-by-year combinations when their GEBV was used. Moreover, phenomic predictions based on GEBVs also led to clearer signals in GWAS analyses, especially in the context of the ridge regression and the artificial neural network. The combination of these methods with GEBVs led to models that identified (almost) the same loci as vegetation index G, but with an increased power. The downside of the ANN is the number of parameters which can be fine-tuned, which again provides flexibility but also potential for overfitting training data. The latter may also have been the main problem for the GBRT approach.

Using GEBVs of traits in a phenomic prediction context may be a valuable path to harness the potential of available data. Moreover, future studies could benefit from incorporating the temporal development of phenomic data (as done by DeSalvio et al. [20]) to capture disease progression dynamics, potentially increasing the biological signal and prediction stability. Finally, for ANN, the setup for model training including the loss function should be considered to explore possibilities to focus on an increase in predictive ability or genetic signal. All data used in this study are publicly available, and we encourage other researchers to retrain or test alternative models on the same datasets.

## CRediT authorship contribution statement

Martini, Johannes Wolfgang Robert: Conceptualization, Formal analysis, Methodology, Writing – original draft, Writing – review & editing.

Montesinos-Lopez, Osval A: Conceptualization, Writing – review & editing.

Crossa, Jose: Conceptualization, Writing – review & editing.

Ortiz, Rodomiro: Conceptualization, Writing – review & editing.

## Data availability

All data used here is readily available for download from the CIMMYT Research Data repository at https://hdl.handle.net/11529/10548898. The access to the genotypic data requires a registration. The data has previously been originally used by Loldaze et al. [21,22]. For more details on which files have been used in this work, please see ‘Materials and Methods - Data’. Estimated GEBVs can be obtained from the corresponding authors.

## Declaration of competing interest

The authors declare that they have no known competing financial interests or personal relationships that could have appeared to influence the work reported in this paper.
