# Supplementary material for: Comparing statistical ‘phenomic prediction’ models for remote-sensing-based phenotyping of maize susceptibility to common rust
Source: Plant Phenomics. 2025 Nov 5;7(4):100134. doi: 10.1016/j.plaphe.2025.100134 (PMC13109342; doi:10.1016/j.plaphe.2025.100134)
Supplement: Multimedia component 1 [file mmc1.docx]

# Supplementary


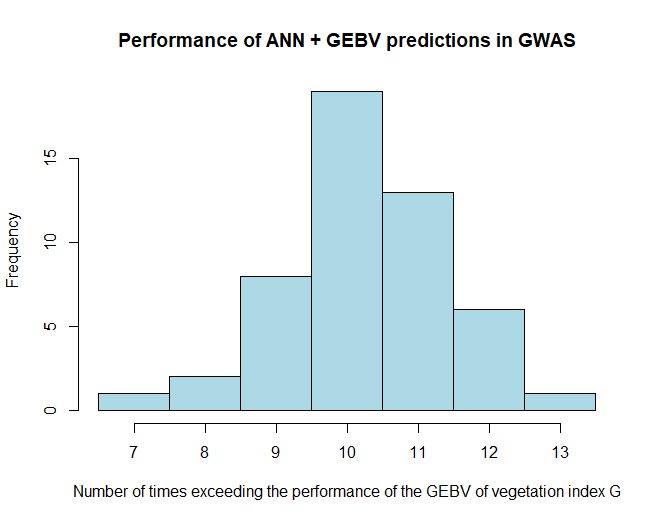


**Supplementary Figure 1** Histogram of the number of times that the predictions with ANN based on GEBVs lead to higher -log(p) values than the GEBV of vegetation index G in a GWAS analysis.

Supplementary Table 1 **Estimated coefficients of model Eq. (1)** when using the different population x year combinations for their estimation based on the BT-OLS model.

| Estimated from | | | | | | |
| --- | --- | --- | --- | --- | --- | --- |
|  | Pop 1, 2019 | Pop 1, 2020 | Pop 2, 2019 | Pop 2, 2020 | Pop 3, 2019 | Pop 3, 2020 |
| $\mu$ | 0 | 0 | 0 | 0 | 0 | 0 |
| $\beta_{\mathrm{gre}}$ | -0.42 | -0.94 | 0 | -0.39 | -0.34 | -0.49 |
| $\beta_{\mathrm{nir}}$ | -0.59 | -0.83 | -0.35 | -0.96 | -0.46 | -0.93 |
| $\beta_{\mathrm{red}}$ | 0.92 | 1.05 | 0.69 | 0.71 | 0.85 | 0.82 |
| $\beta_{\mathrm{reg}}$ | 0.21 | 0.96 | -0.26 | 0.69 | 0.12 | 0.88 |
| $\beta_{\mathrm{thr}}$ | -0.67 | -0.37 | -0.55 | -0.17 | -0.44 | -0.13 |

Supplementary Table 2 **Predictive ability of AT-OLS for the VS.** Correlation of the AT-OLS predictions with the VS of the respective data set and when trained with the data of different population$\times$year combinations. ‘Benchmark’ denotes the highest (absolute) correlation a vegetation index reached with the VS of the respective data set. Correlations equal to or higher than the respective benchmark that is when the AT-OLS prediction outperformed the best vegetation index in terms of correlation to the VS are highlighted in bold. The benchmark correlation has been reached in two out of the 30 cases in which the training and prediction set were not identical. Lin’s Concordance Correlation Coefficient between prediction and the VS is given in brackets.

|  | Predicting to | | | | | | |
| --- | --- | --- | --- | --- | --- | --- | --- |
| Predicting from |  | Pop 1, 2019 | Pop 1, 2020 | Pop 2, 2019 | Pop 2, 2020 | Pop 3, 2019 | Pop 3, 2020 |
|  | Pop 1, 2019 | 0.83 (0.81) | 0.40 (0.40) | 0.71 (0.69) | 0.69 (0.68) | 0.70 (0.68) | 0.61 (0.61) |
|  | Pop 1, 2020 | 0.59 (0.46) | 0.78 (0.76) | 0.44 (0.34) | 0.66 (0.65) | 0.48 (0.35) | 0.63 (0.60) |
|  | Pop 2, 2019 | **0.70** (0.69) | 0.20 (0.11) | 0.83 (0.82) | 0.11 (0.09) | **0.76** (0.75) | 0.32 (0.25) |
|  | Pop 2, 2020 | 0.36 (0.31) | 0.22 (0.15) | 0.19 (0.16) | 0.88 (0.88) | 0.04 (0.03) | 0.52 (0.52) |
|  | Pop 3, 2019 | 0.49 (0.37) | 0.33 (0.10) | 0.73 (0.53) | 0.58 (0.29) | 0.81 (0.80) | 0.70 (0.42) |
|  | Pop 3, 2020 | 0.05 (0.05) | 0.03 (0.01) | -0.15 (-0.12) | 0.34 (0.26) | -0.14 (-0.07) | 0.90 (0.89) |
|  | Benchmark | 0.69 (G) | 0.73 (G) | 0.74 (NDVI) | 0.86 (G) | 0.73 (G) | 0.87 (G) |

Supplementary Table 3 **Predictive ability of BT-RR for the VS.** Correlation of the BT-RR predictions with the VS of the respective data set and when trained with the data of different population$\times$year combinations. ‘Benchmark’ denotes the highest (absolute) correlation a vegetation index reached with the VS of the respective data set. Correlations equal to or higher than the respective benchmark that is when the BT-RR prediction outperformed the best vegetation index in terms of correlation to the VS are highlighted in bold. The benchmark correlation has been reached in 15 out of the 30 cases in which the training and prediction set were not identical. Lin’s Concordance Correlation Coefficient between prediction and the VS is given in brackets.

|  | Predicting to | | | | | | |
| --- | --- | --- | --- | --- | --- | --- | --- |
| Predicting from |  | Pop 1, 2019 | Pop 1, 2020 | Pop 2, 2019 | Pop 2, 2020 | Pop 3, 2019 | Pop 3, 2020 |
|  | Pop 1, 2019 | 0.80 (0.78) | 0.71 (0.68) | **0.78** (0.76) | 0.77 (0.75) | **0.78** (0.77) | 0.76 (0.74) |
|  | Pop 1, 2020 | **0.78** (0.74) | 0.77 (0.74) | 0.71 (0.67) | 0.82 (0.80) | **0.75** (0.71) | 0.81 (0.80) |
|  | Pop 2, 2019 | **0.77** (0.75) | 0.71 (0.66) | 0.81 (0.80) | 0.79 (0.76) | **0.79** (0.78) | 0.77 (0.74) |
|  | Pop 2, 2020 | **0.70** (0.68) | **0.73** (0.71) | **0.77** (0.75) | 0.86 (0.85) | **0.76** (0.74) | 0.85 (0.84) |
|  | Pop 3, 2019 | **0.78** (0.75) | **0.75** (0.70) | **0.80** (0.78) | 0.82 (0.80) | 0.80 (0.78) | 0.81 (0.79) |
|  | Pop 3, 2020 | 0.68 (0.65) | 0.72 (0.71) | **0.75** (0.72) | 0.85 (0.84) | **0.74** (0.71) | 0.86 (0.85) |
|  | Benchmark | 0.69 (G) | 0.73 (G) | 0.74 (NDVI) | 0.86 (G) | 0.73 (G) | 0.87 (G) |

Supplementary Table 4 **Genetic signal in GWAS analyses when using AT-RR predictions as ‘phenomic’ vegetation index.** Genetic signals described by the highest -log(p) value on chromosome 10 and the position of the corresponding marker. Rows provide different traits, that is the VS, the vegetation index G and AT-RR predictions when training a model on different training sets. Training of the model can be interpreted as deriving a phenomic index. Cases in which training and prediction set are identical are highlighted as italic. Cases in which the performance of the vegetation index G was exceeded by the AT-RR model are highlighted as bold. The genetic signal obtained from vegetation index G has been outperformed in 16 out of the 30 cases in which the training and prediction set were not identical.

|  | | **Population 1** | | **Population 2** | | **Population 3** | |
| --- | --- | --- | --- | --- | --- | --- | --- |
|  |  | 2019 | 2020 | 2019 | 2020 | 2019 | 2020 |
| VS | Pos.  $-log(p)$ | 2,954,643  24.77 | 2,954,643  23.69 | 20,858,205  15.98 | 20,858,205  20.10 | 2,954,643  33.32 | 2,954,643  29.33 |
| G | Pos.  $-log(p)$ | 2,954,643  12.16 | 2,639,580  6.88 | 2,954,643  14.75 | 20,858,205  11.40 | 2,954,643  14.19 | 2,954,643  19.04 |
| AT-RR  Pop1 2019 | Pos.  $-log(p$*)* | *2,954,643*  *18.49* | **2,639,580**  **8.55** | **2,954,643**  **15.02** | 20,858,205  8.00 | **2,954,643**  **16.67** | 3,909,507  11.09 |
| AT-RR  Pop1 2020 | Pos.  $-log(p)$ | **2,954,643**  **16.88** | *2,639,580*  *8.71* | **2,954,643**  **15.57** | 20,858,205  11.31 | **2,954,643**  **15.73** | 2,954,643  16.26 |
| AT-RR  Pop2 2019 | Pos.  $-log(p)$ | **2,639,580**  **14.04** | **2,639,580**  **7.04** | *2,954,643*  *16.08* | 20,858,205  7.24 | **2,954,643**  **16.88** | 3,909,507  13.11 |
| AT-RR  Pop2 2020 | Pos.  $-log(p)$ | **2,954,643**  **12.57** | 2,639,580  6.61 | 2,954,643  14.65 | *20,858,205*  *10.92* | **2,954,643**  **15.15** | 2,954,643  18.41 |
| AT-RR  Pop3 2019 | Pos.  $-log(p)$ | **2,368,199**  **16.08** | **2,639,580**  **8.05** | **2,954,643**  **16.15** | 20,858,205  8.42 | *2,954,643*  *17.93* | 3,909,507  14.28 |
| AT-RR  Pop3 2020 | Pos.  $-log(p)$ | **2,954,643**  **12.59** | 2,639,580  6.87 | 2,954,643  13.61 | **20,858,205**  **12.17** | 2,954,643  14.09 | *2,954,643*  *19.22* |

Supplementary Table 5 **Predictive ability of AT-LASSO for the VS.** Average correlation of the AT-LASSO predictions with the VS of the respective data set and when trained with the data of different population$\times$year combinations. Averages are obtained from 50 rounds of determining λ based on cv.glmnet. The ‘Benchmark’ denotes the highest (absolute) correlation a vegetation index reached with the VS of the respective data set. Average correlations equal to or higher than the respective benchmark that is when the AT-LASSO prediction outperformed or equaled the best vegetation index in terms of correlation to the VS are highlighted in bold. The benchmark correlation has been reached in 19 out of the 30 cases in which the training and prediction set were not identical. Average Lin’s Concordance Correlation Coefficients between prediction and the VS are given in brackets.

|  | Predicting to | | | | | | |
| --- | --- | --- | --- | --- | --- | --- | --- |
| Predicting from |  | Pop 1, 2019 | Pop 1, 2020 | Pop 2, 2019 | Pop 2, 2020 | Pop 3, 2019 | Pop 3, 2020 |
|  | Pop 1, 2019 | 0.82 (0.80) | 0.66 (0.65) | **0.76** (0.73) | 0.72 (0.70) | **0.77** (0.76) | 0.73 (0.70) |
|  | Pop 1, 2020 | **0.78** (0.76) | 0.78 (0.75) | **0.75** (0.74) | 0.84 (0.82) | **0.77** (0.76) | 0.85 (0.83) |
|  | Pop 2, 2019 | **0.75** (0.73) | 0.72 (0.70) | 0.82 (0.80) | 0.80 (0.78) | **0.80** (0.78) | 0.77 (0.76) |
|  | Pop 2, 2020 | **0.72** (0.72) | **0.74** (0.74) | **0.75** (0.74) | 0.87 (0.87) | **0.75** (0.75) | **0.88** (0.87) |
|  | Pop 3, 2019 | **0.77** (0.74) | **0.74** (0.72) | **0.81** (0.78) | 0.81 (0.79) | 0.81 (0.79) | 0.80 (0.78) |
|  | Pop 3, 2020 | **0.71** (0.71) | **0.74** (0.74) | 0.70 (0.70) | **0.86** (0.86) | **0.73** (0.73) | 0.89 (0.89) |
|  | Benchmark | 0.69 (G) | 0.73 (G) | 0.74 (NDVI) | 0.86 (G) | 0.73 (G) | 0.87 (G) |

Supplementary Table 6 **Genetic signal in GWAS analyses when using AT-LASSO predictions as ‘phenomic index’.** **Results shown are of one specific iteration**. Genetic signals described by the highest -log(p) value on chromosome 10 and the position of the corresponding marker. Rows provide different traits, that is the VS, the vegetation index G and AT-LASSO predictions when training a model on different training sets. Training of the model can be interpreted as deriving a phenomic index. Cases in which training and prediction set are identical are highlighted as italic. Cases in which the performance of the vegetation index G was exceeded by the AT-LASSO model are highlighted as bold. The genetic signal obtained from vegetation index G has been outperformed in 15 out of the 30 cases in which the training and prediction set were not identical. Across the 50 repetitions, the number of exceeding the performance of G varied between 14 and 18 with average 15.82.

|  | | **Population 1** | | **Population 2** | | **Population 3** | |
| --- | --- | --- | --- | --- | --- | --- | --- |
|  |  | 2019 | 2020 | 2019 | 2020 | 2019 | 2020 |
| VS | Pos.  $-log(p)$ | 2,954,643  24.77 | 2,954,643  23.69 | 20,858,205  15.98 | 20,858,205  20.10 | 2,954,643  33.32 | 2,954,643  29.33 |
| G | Pos.  $-log(p)$ | 2,954,643  12.16 | 2,639,580  6.88 | 2,954,643  14.75 | 20,858,205  11.40 | 2,954,643  14.19 | 2,954,643  19.04 |
| AT-RR  Pop1 2019 | Pos.  $-log(p$*)* | *2,954,643*  *18.25* | 3,909,507  6.71 | 2,954,643  13.96 | 2,216,447  7.10 | **2,954,643**  **16.74** | 3,909,507  10.75 |
| AT-RR  Pop1 2020 | Pos.  $-log(p)$ | **2,954,643**  **17.17** | *2,639,580*  *8.75* | **2,954,643**  **15.43** | 20,858,205  11.26 | **2,954,643**  **15.52** | 2,954,643  16.04 |
| AT-RR  Pop2 2019 | Pos.  $-log(p)$ | **2,639,580**  **13.91** | **2,639,580**  **6.99** | *2,954,643*  *15.71* | 20,858,205  7.07 | **2,954,643**  **16.90** | 3,909,507  13.07 |
| AT-RR  Pop2 2020 | Pos.  $-log(p)$ | **2,954,643**  **12.79** | 2,639,580  6.76 | 2,954,643  14.45 | *20,858,205*  *11.13* | **2,954,643**  **15.34** | 2,954,643  18.55 |
| AT-RR  Pop3 2019 | Pos.  $-log(p)$ | **2,368,199**  **15.33** | **2,639,580**  **7.75** | **2,954,643**  **15.81** | 20,858,205  7.99 | *2,954,643*  *17.85* | 2,954,643  13.98 |
| AT-RR  Pop3 2020 | Pos.  $-log(p)$ | **2,954,643**  **13.46** | 2,639,580  6.83 | 2,954,643  13.12 | **20,858,205**  **12.23** | **2,954,643**  **15.66** | *2,954,643*  *19.32* |

Supplementary Table 7 **Predictive ability of a neural network with torch and ReLU activation function.** Means of 50 different training processes are shown (training is not deterministic, which generates variation). Average Lin’s Concordance Correlation Coefficients between prediction and the VS are given in brackets.

|  | Predicting to | | | | | | | |
| --- | --- | --- | --- | --- | --- | --- | --- | --- |
| Predicting from |  | Pop 1, 2019 | Pop 1, 2020 | Pop 2, 2019 | Pop 2, 2020 | Pop 3, 2019 | Pop 3, 2020 |  |
|  | Pop 1, 2019 | 0.81 (0.80) | 0.72 (0.70) | **0.76** (0.75) | 0.77 (0.76) | **0.78** (0.78) | 0.78 (0.77) |  |
|  | Pop 1, 2020 | **0.77** (0.75) | 0.79 (0.76) | **0.77** (0.75) | 0.84 (0.83) | **0.77** (0.76) | 0.85 (0.84) |  |
|  | Pop 2, 2019 | **0.76** (0.74) | 0.72 (0.71) | 0.83 (0.81) | 0.82 (0.81) | **0.79** (0.78) | 0.79 (0.78) |  |
|  | Pop 2, 2020 | **0.69** (0.68) | **0.73** (0.72) | **0.75** (0.75) | 0.88 (0.87) | **0.74** (0.73) | **0.88** (0.87) |  |
|  | Pop 3, 2019 | **0.78** (0.76) | 0.72 (0.70) | **0.81** (0.79) | 0.83 (0.82) | 0.82 (0.80) | 0.81 (0.80) |  |
|  | Pop 3, 2020 | 0.66 (0.65) | 0.72 (0.71) | 0.71 (0.70) | **0.87** (0.86) | 0.69 (0.69) | 0.89 (0.89) |  |
|  | Benchmark | 0.69 (G) | 0.73 (G) | 0.74 (NDVI) | 0.86 (G) | 0.73 (G) | 0.87 (G) |  |

Supplementary Table 8 **Predictive ability of a neural network with torch and sigmoid activation function.** Means of 50 different training processes are shown (training is not deterministic, which generates variation). Average Lin’s Concordance Correlation Coefficients between prediction and the VS are given in brackets.

|  | Predicting to | | | | | | |
| --- | --- | --- | --- | --- | --- | --- | --- |
| Predicting from |  | Pop 1, 2019 | Pop 1, 2020 | Pop 2, 2019 | Pop 2, 2020 | Pop 3, 2019 | Pop 3, 2020 |
|  | Pop 1, 2019 | 0.79 (0.74) | 0.71 (0.65) | **0.78** (0.74) | 0.82 (0.78) | **0.79** (0.75) | 0.81 (0.77) |
|  | Pop 1, 2020 | **0.69** (0.66) | 0.76 (0.71) | **0.77** (0.74) | **0.86** (0.82) | **0.75** (0.72) | 0.86 (0.83) |
|  | Pop 2, 2019 | **0.72** (0.69) | 0.71 (0.67) | 0.79 (0.77) | 0.85 (0.82) | **0.78** (0.75) | 0.83 (0.81) |
|  | Pop 2, 2020 | 0.63 (0.63) | 0.70 (0.69) | **0.74** (0.73) | 0.86 (0.85) | 0.71 (0.70) | 0.86 (0.86) |
|  | Pop 3, 2019 | **0.74** (0.72) | 0.69 (0.66) | **0.79** (0.76) | 0.84 (0.81) | 0.79 (0.76) | 0.82 (0.80) |
|  | Pop 3, 2020 | 0.61 (0.60) | 0.69 (0.67) | 0.72 (0.71) | 0.85 (0.84) | 0.68 (0.67) | 0.87 (0.86) |
|  | Benchmark | 0.69 (G) | 0.73 (G) | 0.74 (NDVI) | 0.86 (G) | 0.73 (G) | 0.87 (G) |

Supplementary Table 9 **Predictive ability of AT-GBRT for the VS.** Correlation of the AT-GBRT predictions with the VS of the respective data set and when trained with the data of different population$\times$year combinations. ‘Benchmark’ denotes the highest (absolute) correlation a vegetation index reached with the VS of the respective data set. Correlations equal to or higher than the respective benchmark that is when the AT-GBRT prediction outperformed the best vegetation index in terms of correlation to the VS are highlighted in bold. The benchmark correlation has been reached in 8 out of the 30 cases in which the training and prediction set were not identical. Lin’s Concordance Correlation Coefficient between prediction and the VS is given in brackets.

|  | Predicting to | | | | | | |
| --- | --- | --- | --- | --- | --- | --- | --- |
| Predicting from |  | Pop 1, 2019 | Pop 1, 2020 | Pop 2, 2019 | Pop 2, 2020 | Pop 3, 2019 | Pop 3, 2020 |
|  | Pop 1, 2019 | 0.87 (0.83) | 0.69 (0.61) | **0.74** (0.70) | 0.78 (0.72) | **0.77** (0.73) | 0.80 (0.75) |
|  | Pop 1, 2020 | **0.69** (0.65) | 0.86 (0.81) | 0.73 (0.67) | 0.82 (0.78) | **0.73** (0.67) | 0.83 (0.80) |
|  | Pop 2, 2019 | **0.72** (0.69) | 0.69 (0.62) | 0.88 (0.84) | 0.81 (0.75) | **0.78** (0.74) | 0.80 (0.76) |
|  | Pop 2, 2020 | 0.64 (0.61) | 0.71 (0.68) | 0.73 (0.70) | 0.90 (0.88) | 0.71 (0.68) | 0.86 (0.85) |
|  | Pop 3, 2019 | **0.73** (0.71) | 0.65 (0.60) | **0.79** (0.76) | 0.80 (0.76) | 0.87 (0.83) | 0.81 (0.78) |
|  | Pop 3, 2020 | 0.67 (0.65) | 0.68 (0.67) | 0.71 (0.69) | 0.84 (0.82) | 0.71 (0.68) | 0.92 (0.90) |
|  | Benchmark | 0.69 (G) | 0.73 (G) | 0.74 (NDVI) | 0.86 (G) | 0.73 (G) | 0.87 (G) |

Supplementary Table 10 **Genetic signal in GWAS analyses when using AT-GBRT predictions as ‘phenomic index’.**  Genetic signals described by the highest -log(p) value on chromosome 10 and the position of the corresponding marker. Rows provide different traits, that is the VS, the vegetation index G and AT-GBRT predictions when training a model on different training sets. Cases in which training and prediction set are identical are highlighted as italic. Cases in which the performance of the vegetation index G was exceeded by the AT-GBRT method are highlighted as bold. The genetic signal obtained from vegetation index G has been outperformed in 13 out of the 30 cases in which the training and prediction set were not identical.

|  | | **Population 1** | | **Population 2** | | **Population 3** | |
| --- | --- | --- | --- | --- | --- | --- | --- |
|  |  | 2019 | 2020 | 2019 | 2020 | 2019 | 2020 |
| VS | Pos.  $-log(p)$ | 2,954,643  24.77 | 2,954,643  23.69 | 20,858,205  15.98 | 20,858,205  20.10 | 2,954,643  33.32 | 2,954,643  29.33 |
| G | Pos.  $-log(p)$ | 2,954,643  12.16 | 2,639,580  6.88 | 2,954,643  14.75 | 20,858,205  11.40 | 2,954,643  14.19 | 2,954,643  19.04 |
| AT-GBRT  Pop1 2019 | Pos.  $-log(p$) | *2,954,643*  *19.46* | 2,639,580  6.81 | 20,858,205  13.64 | 20,858,205  9.52 | **2,954,643**  **16.39** | 2,954,643  16.80 |
| AT-GBRT  Pop1 2020 | Pos.  $-log(p$) | **2,954,643**  **12.95** | *2,639,580*  *11.60* | **20,858,205**  **14.85** | 20,858,205  8.96 | **2,954,643**  **16.35** | 2,954,643  16.95 |
| AT-GBRT  Pop2 2019 | Pos.  $-log(p$) | **2,954,643**  **13.53** | **2,639,580**  **6.98** | *2,954,643*  *19.44* | 2,017,344  10.00 | **2,954,643**  **20.07** | 2,954,643  16.23 |
| AT-GBRT  Pop2 2020 | Pos.  $-log(p$) | 2,639,580  10.09 | 2,639,580  5.71 | 2,954,643  14.27 | *20,858,205*  *13.77* | **2,954,643**  **16.73** | **2,954,643**  **19.53** |
| AT-GBRT  Pop3 2019 | Pos.  $-log(p$) | **2,954,643**  **14.20** | 2,639,580  5.80 | **2,954,643**  **16.01** | 2,954,643  10.47 | *2,954,643*  *25.18* | 2,954,643  17.55 |
| AT-GBRT  Pop3 2020 | Pos.  $-log(p$) | 2,954,643  11.84 | 2,639,580  5.17 | 2,954,643  13.02 | **20,858,205**  **13.14** | **2,954,643**  **14.69** | *2,954,643*  *23.48* |

Supplementary Table 11 **Predictive ability of predicting genomically adjusted** ${\hat{\boldsymbol{VS}}}_{\boldsymbol{K}}$ **by genomically adjusted** ${\hat{\boldsymbol{P}}}_{\boldsymbol{K}}$ **with an AT-RR approach.** Correlation of the AT-RR predictions with the $\hat{VS}_{K}$ of the respective data set and when trained with the data of different population$\times$year combinations. ‘Benchmark’ denotes the highest (absolute) correlation a vegetation index reached with the VS of the respective data set. Correlations equal to or higher than the respective benchmark are highlighted in bold. The benchmark correlation has been reached in 19 out of the 30 cases in which the training and prediction set were not identical.

|  | Predicting to | | | | | | |
| --- | --- | --- | --- | --- | --- | --- | --- |
| Predicting from |  | Pop 1, 2019 | Pop 1, 2020 | Pop 2, 2019 | Pop 2, 2020 | Pop 3, 2019 | Pop 3, 2020 |
|  | Pop 1, 2019 | 0.89 (0.89) | 0.70 (0.68) | **0.79** (0.79) | 0.74 (0.74) | **0.77** (0.77) | 0.80 (0.79) |
|  | Pop 1, 2020 | **0.85** (0.85) | 0.82 (0.80) | **0.80** (0.79) | 0.83 (0.83) | **0.83** (0.80) | **0.88** (0.88) |
|  | Pop 2, 2019 | **0.84** (0.81) | 0.70 (0.62) | 0.88 (0.87) | 0.77 (0.73) | **0.81** (0.81) | 0.80 (0.77) |
|  | Pop 2, 2020 | **0.79** (0.77) | **0.74** (0.74) | 0.63 (0.61) | 0.90 (0.89) | **0.81** (0.80) | **0.90** (0.90) |
|  | Pop 3, 2019 | **0.83** (0.80) | **0.75** (0.70) | 0.73 (0.71) | 0.85 (0.82) | 0.84 (0.83) | **0.88** (0.84) |
|  | Pop 3, 2020 | **0.80** (0.79) | **0.79** (0.79) | **0.75** (0.73) | 0.84 (0.84) | **0.83** (0.81) | 0.93 (0.93) |
|  | Benchmark | 0.69 (G) | 0.73 (G) | 0.74 (NDVI) | 0.86 (G) | 0.73 (G) | 0.87 (G) |

Supplementary Table 12 **Predictive ability of predicting genomically adjusted** ${\hat{\boldsymbol{VS}}}_{\boldsymbol{K}}$ **by genomically adjusted** ${\hat{\boldsymbol{P}}}_{\boldsymbol{K}}$ **with an ANN approach.** Mean correlation of the ANN predictions (50 reps) with the $\hat{VS}_{K}$ of the respective data set and when trained with the data of different population$\times$year combinations. ‘Benchmark’ denotes the highest (absolute) correlation a vegetation index reached with the VS of the respective data set. Correlations equal to or higher than the respective benchmark are highlighted in bold. The benchmark correlation has been reached in 19 out of the 30 cases in which the training and prediction set were not identical. Average Lin’s Concordance Correlation Coefficients between prediction and the VS are given in brackets.

|  | Predicting to | | | | | | |
| --- | --- | --- | --- | --- | --- | --- | --- |
| Predicting from |  | Pop 1, 2019 | Pop 1, 2020 | Pop 2, 2019 | Pop 2, 2020 | Pop 3, 2019 | Pop 3, 2020 |
|  | Pop 1, 2019 | 0.88 (0.88) | **0.75** (0.73) | 0.71 (0.71) | 0.77 (0.77) | **0.79** (0.78) | 0.83 (0.83) |
|  | Pop 1, 2020 | **0.83** (0.82) | 0.80 (0.78) | 0.71 (0.70) | 0.85 (0.84) | **0.81** (0.81) | **0.87** (0.87) |
|  | Pop 2, 2019 | **0.81** (0.79) | 0.71 (0.67) | 0.86 (0.85) | 0.79 (0.77) | **0.80** (0.80) | 0.81 (0.79) |
|  | Pop 2, 2020 | **0.76** (0.76) | **0.76** (0.75) | 0.73 (0.73) | 0.89 (0.88) | **0.80** (0.80) | **0.89** (0.89) |
|  | Pop 3, 2019 | **0.83** (0.81) | **0.73** (0.70) | **0.76** (0.74) | 0.83 (0.81) | 0.85 (0.84) | 0.85 (0.83) |
|  | Pop 3, 2020 | **0.76** (0.76) | **0.76** (0.75) | **0.74** (0.73) | **0.88** (0.87) | **0.80** (0.80) | 0.91 (0.90) |
|  | Benchmark | 0.69 (G) | 0.73 (G) | 0.74 (NDVI) | 0.86 (G) | 0.73 (G) | 0.87 (G) |

Supplementary Table 13 **Predictive ability of predicting genomically adjusted** ${\hat{\boldsymbol{VS}}}_{\boldsymbol{K}}$ **by genomically adjusted** ${\hat{\boldsymbol{P}}}_{\boldsymbol{K}}$ **with an AT-GBRT approach.** Correlation of the AT-GBRT predictions with the $\hat{VS}_{K}$ of the respective data set and when trained with the data of different population$\times$year combinations. ‘Benchmark’ denotes the highest (absolute) correlation a vegetation index reached with the VS of the respective data set. Correlations equal to or higher than the respective benchmark are highlighted in bold. The benchmark correlation has been reached in 19 out of the 30 cases in which the training and prediction set were not identical.

|  | Predicting to | | | | | | |
| --- | --- | --- | --- | --- | --- | --- | --- |
| Predicting from |  | Pop 1, 2019 | Pop 1, 2020 | Pop 2, 2019 | Pop 2, 2020 | Pop 3, 2019 | Pop 3, 2020 |
|  | Pop 1, 2019 | 0.91 (0.88) | 0.72 (0.66) | **0.74** (0.71) | 0.81 (0.77) | **0.82** (0.80) | 0.86 (0.83) |
|  | Pop 1, 2020 | **0.80** (0.75) | 0.86 (0.81) | **0.74** (0.69) | 0.85 (0.81) | **0.82** (0.77) | **0.89** (0.85) |
|  | Pop 2, 2019 | **0.79** (0.75) | 0.71 (0.62) | 0.90 (0.86) | 0.80 (0.74) | **0.80** (0.76) | 0.82 (0.77) |
|  | Pop 2, 2020 | **0.75** (0.73) | **0.73** (0.71) | **0.74** (0.72) | 0.92 (0.90) | **0.80** (0.78) | **0.90** (0.89) |
|  | Pop 3, 2019 | **0.78** (0.75) | 0.71 (0.64) | **0.75** (0.72) | 0.80 (0.76) | 0.89 (0.87) | 0.84 (0.80) |
|  | Pop 3, 2020 | **0.77** (0.75) | **0.73** (0.72) | 0.73 (0.71) | **0.87** (0.85) | **0.81** (0.79) | 0.94 (0.92) |
|  | Benchmark | 0.69 (G) | 0.73 (G) | 0.74 (NDVI) | 0.86 (G) | 0.73 (G) | 0.87 (G) |
